# Impurity Doping in Mg(OH)_2_ for n-Type and p-Type Conductivity Control

**DOI:** 10.3390/ma13132972

**Published:** 2020-07-03

**Authors:** Masaya Ichimura

**Affiliations:** Department of Electrical and Mechanical Engineering, Nagoya Institute of Technology, Nagoya 466-8555, Japan; ichimura.masaya@nitech.ac.jp

**Keywords:** Mg(OH)_2_, impurity doping, first-principles calculations, valence control

## Abstract

Magnesium hydroxide (Mg(OH)_2_) has a wide bandgap of about 5.7 eV and is usually considered an insulator. In this study, the energy levels of impurities introduced into Mg(OH)_2_ are predicted by first-principles calculations. A supercell of brucite Mg(OH)_2_ consisting of 135 atoms is used for the calculations, and an impurity atom is introduced either at the substitutional site replacing Mg or the interlayer site. The characteristics of impurity levels are predicted from density-of-states analysis for the charge-neutral cell. According to the results, possible shallow donors are trivalent cations at the substitutional site (e.g., Al and Fe) and cation atoms at the interlayer site (Cu, Ag, Na, and K). On the other hand, an interlayer F atom can be a shallow acceptor. Thus, valence control by impurity doping can turn Mg(OH)_2_ into a wide-gap semiconductor useful for electronics applications.

## 1. Introduction

Transparent conductive materials (TCMs), which are indispensable for transparent electronics, have been widely investigated [1]. TCMs are used as conductors for electrodes in displays, touch panels, and solar cells, and as active semiconductor layers in thin-film transistors (TFTs). To achieve transparency toward visible light, a TCM must have a bandgap larger than 3 eV. Many oxides act as TCMs, and they are known as transparent conductive oxides (TCO) [2]; In_2_O_3_ and SnO_2_ are the most popular materials for transparent electrodes. Sulfides and oxy-chalcogenides have also attracted attention as TCMs [3]. The majority of TCMs show n-type conductivity without intentional doping, and conductivity can be controlled relatively easily by doping with a proper donor, e.g., Sn for In_2_O_3_ (ITO) and F for SnO_2_ (FTO). For ZnO, Al and Ga are known to be good shallow donors. On the other hand, a limited number of p-type TCMs are known, and it is generally difficult to enhance p-type conductivity by acceptor doping because of self-compensation, deep acceptor level, and the localized nature of holes [3,4,5].

In addition to TCMs, several materials with a bandgap larger than 3 eV have been extensively studied for applications in power electronics and UV optoelectronics. A great number of devices based on diamond, GaN, and SiC (6H, 4H) have been successfully developed. Ga_2_O_3_ is also considered a promising compound for high-power and deep-UV devices. When using these materials for devices, single crystals are required. However, in transparent electronics, polycrystalline or amorphous TCMs deposited on glass or plastic substrates are commonly used.

Ga_2_O_3_ has a bandgap of about 5 eV, and an alloy with Al_2_O_3_ ((Al*_x_*Ga_1−*x*_)_2_O_3_), which has an even larger bandgap, has also been used to fabricate heterostructure devices [6,7,8]. Similarly, the bandgap of ZnO has been enlarged by alloying with MgO (Zn_1−*x*_Mg*_x_*O) [9,10,11]. Thus, since Al_2_O_3_ and MgO are constituents of the alloys used for heterostructure devices, they can also be regarded as useful semiconductor materials. Diamond, with a bandgap of 5.7 eV, becomes semiconducting with proper impurity doping and is used for electronic devices. The possibility of controlling the conductivity by impurity doping has also been examined for SiO_2_ and Al_2_O_3_ based on first-principles calculations [12,13]. In addition, it was reported that Cu-doped amorphous AlO_x_ thin films have p-type conductivity and that the non-doped AlO*_x_*/Cu-doped AlO*_x_* homojunction shows rectification properties [14]. Thus, even materials with a bandgap larger than 5 eV have been investigated as semiconductors.

In this study, first-principles calculations are carried out to investigate the possibility of controlling the conductivity of magnesium hydroxide (Mg(OH)_2_) by impurity doping. Mg(OH)_2_ has a bandgap of 5.7 eV [15,16] and is considered an insulator. However, as mentioned above, materials with a comparable bandgap have already been utilized as semiconductors. Therefore, Mg(OH)_2_ may also be useful for electronic devices as a wide-gap semiconductor. MgO is unstable in air (deliquesces), i.e., it easily reacts with water molecules; however, Mg(OH)_2_, the product of the reaction of MgO with water, is stable in air. Mg(OH)_2_ is decomposed to MgO by annealing in a dry atmosphere at the conversion temperature of 350–400 °C [16,17]. Accordingly, Mg(OH)_2_ is thermally stable at room temperature; if its electrical properties could be controlled, it would find many applications in electronics as a wide-gap semiconductor. In fact, a few research groups have attempted to use Mg(OH)_2_ thin films in heterostructure solar cells [18,19] and dye-sensitized solar cells [20,21]. However, in those works, impurity doping was not attempted. Recently, our group reported that semiconducting Cu-doped Mg(OH)_2_ thin films can be obtained by electrochemical deposition [22,23]. Potentiostatically deposited films have an n-type conduction, which is converted to p-type by annealing in air. We also reported preliminary results of first-principles calculations of Cu impurity levels in Mg(OH)_2_, showing that Cu will be a shallow donor if introduced at the interlayer site and a deep acceptor at the substitutional (Mg) site [22]. In this study, impurity levels are calculated for various elements, and the possibility of valence control of Mg(OH)_2_ is analyzed. Since it was previously demonstrated that Mg(OH)_2_ with a large content of carbon is transparent and conductive [24,25,26,27], the impurity levels introduced by carbon are also examined, and the possible origin of large conductivity is discussed.

## 2. Calculation

**Figure 1:**
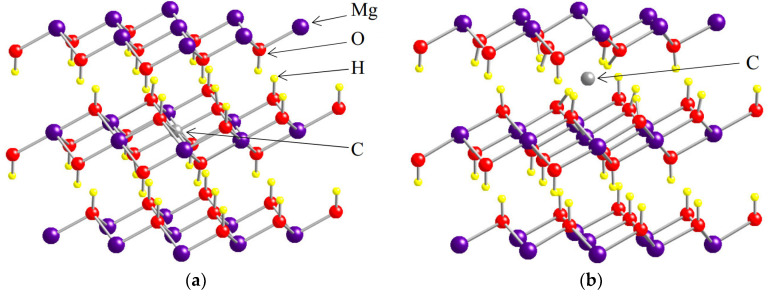
Atomic site of impurity atoms in Mg(OH)_2_, with C used as an example. (**a**) The substitutional (Mg) site; (**b**) the interlayer site.

Mg(OH)_2_ has a layered brucite structure where Mg atoms are octahedrally coordinated to six OH groups. To investigate impurity levels, a 3 × 3 × 3 supercell consisting of 135 atoms was adopted, and one impurity atom was introduced in the supercell. As in the previous study, the substitutional (Mg) site and interlayer site are considered. Figure 1 uses carbon as an example to show an impurity atom introduced at the substitutional site (Figure 1a) and interlayer site (Figure 1b). Hereafter, an impurity atom located at the substitutional site is denoted as “s-C”, and an impurity atom located at the interlayer site is denoted as “i-C” (again using carbon as an example).

Table 1 lists the elements used as impurities in the calculations. An atom at the substitutional site is bonded to OH groups. Therefore, the elements that form hydroxides were considered for their substitutional impurities. For the interlayer impurities, elements with a strong ionization tendency, either positive (Na, K) or negative (F, Cl), were considered because an interlayer atom is expected to be an isolated ion between Mg(OH)_2_ layers. For Cu, both substitutional and interlayer sites were considered [22]. For Ag, only the interlayer site was considered because its hydroxide is known to be unstable. In addition, energy levels were also calculated for two native defects: Mg vacancy (V_Mg_) and OH vacancy (V_OH_).

PHASE software (ver. 11.00, University of Tokyo, Tokyo, Japan) was used for the first-principles calculations based on the density-functional theory (DFT). The pseudopotential method was used with the generalized-gradient approximation (GGA) [28]. Brillouin zone integration was carried out using Monkhorst-Pack k-point grids of 3 × 3 × 3. The kinetic energy cutoff of the basis set was 272 eV (20 Rydberg). The atom positions in the supercell were optimized to minimize the total energy, but the lattice constant values were fixed based on reported experimental data (*a* = 0.314 nm, *c* = 0.477 nm). The force convergence criterion for the lattice relaxation was 5 × 10^−2^ eV/Å. To analyze the impurity levels, the density of states (DOS) was calculated for the neutral cell. Since the lattice relaxation accompanying ionization was not considered in the DOS calculation, accurate energy levels could not be obtained. However, the calculation is useful for the preliminary screening of potential donors and acceptors.

## 3. Results and Discussion

### 3.1. Vacancy Defects

**Figure 2:**
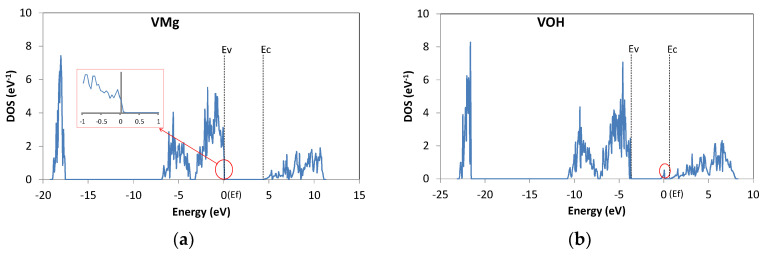
Density of states (DOS) of Mg(OH)_2_ with (**a**) Mg vacancy V_Mg_ and (**b**) OH vacancy V_OH_.

Figure 2 shows the DOS for one Mg(OH)_2_ primitive cell (per eV) for the supercell with V_Mg_ or with V_OH_. The origin of the horizontal axis corresponds to the energy of the highest occupied state, i.e., the Fermi level E_f_. The approximate positions of the conduction band bottom E_c_ and the valence band top E_v_ are highlighted in the plots. It is known that the bandgap is underestimated in the DFT calculation based on GGA. Accordingly, the calculated bandgap of Mg(OH)_2_ is about 4 eV, which is considerably smaller than the experimental value (5.7 eV). Therefore, the depth of the impurity levels will also be underestimated in the following calculations.

In compounds, a cation vacancy tends to act as an acceptor, and an anion vacancy tends to act as a donor. In the DOS spectrum in Figure 2a, there is an empty state at the top of the valence band E_v_, and thus V_Mg_ acts as a shallow acceptor, generating a hole. In contrast, V_OH_ introduces a partially (1/2) occupied energy level in the upper half of the bandgap. However, the material cannot be highly conductive at room temperature (RT) because the energy difference from the conduction band bottom E_c_ is about 1 eV, i.e., V_OH_ is a deep donor. Deep levels introduced by the defects and impurities are summarized in Table 2.

### 3.2. Substitutional Impurities

**Figure 3:**
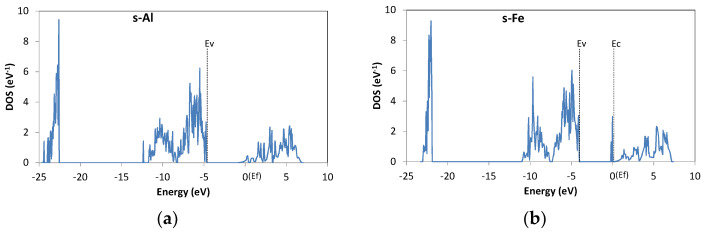
DOS of Mg(OH)_2_ with a substitutional impurity. (**a**) Al; (**b**) Fe.

**Figure 4:**
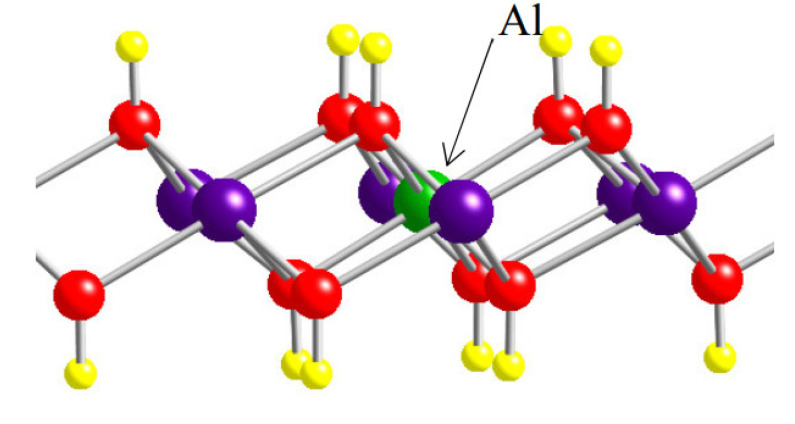
Atomic structure around an s-Al atom after relaxation.

The behavior of impurities at substitutional sites is basically determined by the valence of the impurity, as usually seen for semiconductor doping. The results for s-Al are shown in Figure 3a. Since Al is trivalent, it is expected to act as a donor at the Mg site. In fact, E_f_ seems to be located within the conduction band. There is one electron (per supercell) in the upper band; according to the analysis of local density of states, it is weakly localized around the O atoms bonded to Al. However, the DOS spectrum of the upper band is almost continuous; thus, Al-doped Mg(OH)_2_ would be expected to have n-type conductivity. E_c_ is not shown in the plot because it cannot be precisely defined (due to the weakly localized nature of the lowest-energy states of the upper band). Figure 4 shows the atomic structure around s-Al. No significant lattice relaxation was observed.

The group IB element Cu introduces a partially occupied level at about 1.5 eV above E_v_ (E_v_ + 1.5). Thus, s-Cu is a deep acceptor. Experimentally, Cu-doped Mg(OH)_2_ was found to become highly resistive and p-type after annealing at 400 °C [22]. According to X-ray photoelectron spectroscopy results, the oxidation state of Cu was +2, as in Cu(OH)_2_, after the annealing. This indicates that in annealed Mg(OH)_2_, Cu atoms occupy the substitutional site, bonding to OH groups and becoming deep acceptors, as suggested by the present calculations. Group IA elements Na and K at the substitutional site would become shallow acceptors, creating one hole, as expected from the valence number. For group II elements Ca and Zn, which have the same valence as Mg, no gap states were introduced. The group IV elements Si and Sn introduced occupied deep levels in the bandgap. As shown in Table 2, the s-Si level is at E_c_ − 1.1 eV, and thus s-Si is a deep donor. The s-Sn level is more than 2 eV away from E_c_, and s-Sn cannot generate conduction electrons.

Figure 3b shows the DOS for s-Fe, which is an example of a trivalent transition metal element. There is a DOS peak adjacent to E_c_, and those states are occupied. Thus, Fe would act as a shallow donor. Similarly, s-Mn and s-Co introduce partially occupied states near E_c_ and would be shallow donors. These transition metal elements can have different oxidation states, including +3. The results show that Fe, Mn, and Co are trivalent at the Mg site and act as a donor. In contrast, s-Ni, which is another transition metal, introduces deep levels (occupied states more than 1 eV away from the band edges), as shown in Table 2. Therefore, Ni will not act as a shallow donor in Mg(OH)_2_. Ni can also be trivalent, but only in a few compounds, and its most common oxidation state is +2. The results indicate that Ni is not a trivalent atom in Mg(OH)_2_.

A mixed hydroxide of Mg and a trivalent element is called layered double hydroxide (LDH) and can be found in natural minerals [29,30,31]. In usual LDHs, hydroxide layers are positively charged due to the trivalent cations, and the positive charge is compensated for by anions in the interlayer. Thus, in LDHs, the trivalent cation donors are compensated for by the interlayer anion acceptors, and therefore LDHs are not conductive. According to the present calculations, if the interlayer anions are removed, the positive charge due to the trivalent cation will be balanced by conduction electrons. It should be noted that this would only be possible for a low concentration of impurities. Here, we consider a small concentration of impurities (which is typical in semiconductor doping). In LDH, a significant fraction (over 10%) of Mg atoms are replaced with other elements; such a large amount of cationic positive charge cannot be balanced by conduction electrons but can only be balanced by interlayer anions.

Na and K are monovalent cations and are expected to act as shallow acceptors if they occupy the Mg site. However, Na is known to migrate from one interstitial site to another (interstitial diffusion) in many materials. Although Na and K form hydroxides, their solubility in water is large; thus, during a chemical synthesis, NaOH or KOH cannot be expected to precipitate and be included in Mg(OH)_2_. As such, it is not certain that Na and K can actually occupy the Mg site.

### 3.3. Interlayer Impurities

**Figure 5:**
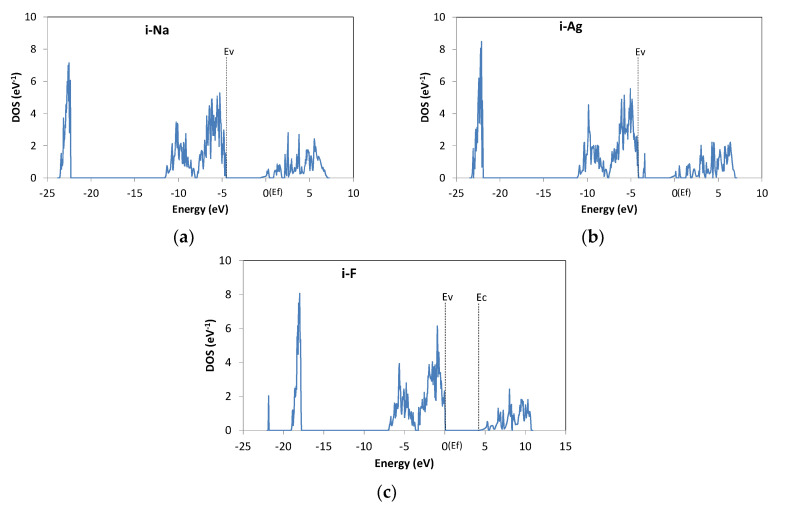
DOS of Mg(OH)_2_ with an interlayer impurity. (**a**) Na; (**b**) Ag; (**c**) F.

As mentioned above, ions can be incorporated into the Mg(OH)_2_ interlayer. The behavior of an interlayer impurity depends on whether it is a cation or anion. Figure 5a shows the DOS for i-Na. E_f_ is located in the conduction band side, and thus the material can be considered to possess n-type conductivity. Similar results were obtained for i-K. The DOS spectrum for i-Ag is shown in Figure 5b. E_f_ is in the upper band, as seen for i-Na and i-K, but there is a peak near E_v_. These states are due to the d orbital of Ag and are fully occupied, i.e., they do not act as acceptor states. i-Cu is also a donor, with occupied deep levels. Thus, the cation atoms act as donors in the interlayer. Experimental results show that Cu-doped Mg(OH)_2_ has n-type conductivity when the oxidation state of Cu is dominantly +1 [22]. While the oxidation state of s-Cu is +2, that of i-Cu will be +1. Thus, the experimental results indicate that i-Cu is actually a donor.

Anionic F and Cl act as acceptors. Figure 5c shows the results for i-F, where there is an empty state (hole) in the valence band. For i-Cl, the gap states are introduced at about 0.5 eV from E_v_. These states are localized around i-Cl and partially occupied; therefore, i-Cl can be regarded as a deep acceptor.

**Figure 6:**
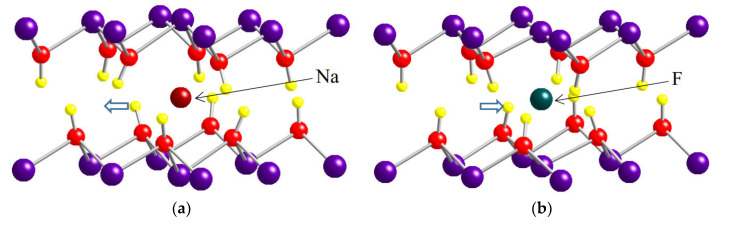
Atomic structure around an interlayer impurity: (**a**) Na; (**b**) F.

Figure 6 shows the lattice relaxation around i-Na and i-F. As indicated by the arrows, H atoms, which are positively charged, are repelled from i-Na and attracted by i-F, as expected from the polarity of charge of the impurity atoms.

### 3.4. Carbon

**Figure 7:**
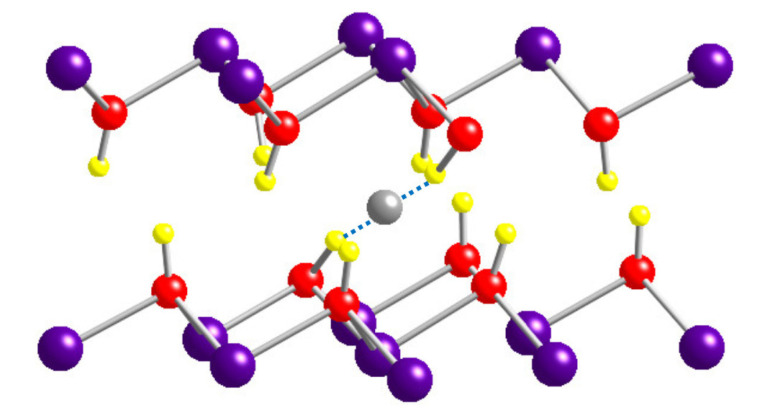
Atomic structure around interlayer C.

As noted in the introduction section, it was reported that Mg(OH)_2_ with a large content of C has large conductivity. The energy levels introduced by i-C and s-C are listed in Table 2. The levels in the lower half of the bandgap are full, while those in the upper half are empty. Thus, those levels cannot act as donors or acceptors. Figure 7 shows the lattice relaxation around i-C. Two of the H atoms around i-C are attracted to C, as denoted by the dashed line.

Murakami et al. speculated that C atoms replace H and bond to O [25]. Accordingly, the DOS was calculated for C bonded to O (O–C). Partially occupied states were created by O–C at E_c_ − 0.7 eV. Therefore, O–C will be a deep donor and cannot induce large conductivity at RT. The authors also reported that O–C introduced half-filled states near the middle of the energy gap. Their work was based on molecular-orbital quantum chemical calculations and was not for crystals but for atom clusters. This could be the main reason for the differences observed in the present work.

**Figure 8:**
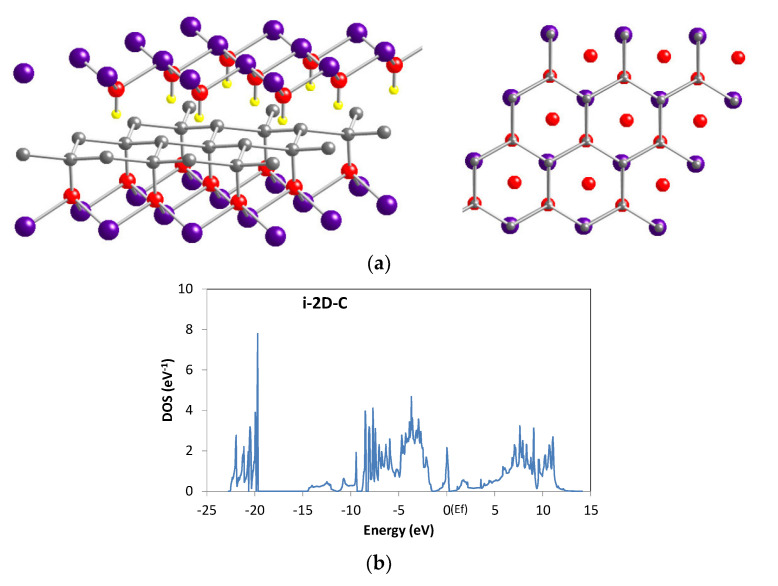
(**a**) Atomic structure of a 2D-C layer in Mg(OH)_2_. (**b**) DOS of Mg(OH)_2_ with a 2D-C layer.

According to the present results, isolated C atoms would not induce significant conduction. However, the C content in the reported conductive C-Mg(OH)_2_ is very large (more than several percent), and the signal due to C–C bonding in X-ray photoelectron spectroscopy had an intensity comparable to that of C–O bonding [26]. Thus, assuming that C forms a two-dimensional (2D) graphite-like structure in Mg(OH)_2_, the DOS was calculated for the structure shown in Figure 8a. C atoms in the interlayer form six-membered rings as in graphite. The lattice constant of the 2D-C layer was expanded to match that of Mg(OH)_2_; hence, the C–C distance is 0.18 nm, which is larger than that of graphite (0.14 nm). In the interlayer, half of the C atoms are located above the Mg atoms, as illustrated in Figure 8a (view along [001] direction). The other half of the C atoms are placed above O atoms, replacing the H atoms. The DOS for 2D-C is shown in Figure 8b. A band is formed within the Mg(OH)_2_ bandgap, and E_f_ is in that band, i.e., the material is metallic. Therefore, the high conductivity reported for C-Mg(OH)_2_ is likely due to a network of such carbon 2D structures.

### 3.5. Summary

The results of this work are summarized in Table 3. Levels with an energy difference from the band edge (E_a_) smaller than 0.5 eV are tentatively classified as “shallow”. Levels with E_a_ between 0.5 and 1.0 eV are categorized as “deep donor or acceptor”, and those with E_a_ > 1.0 eV are classified as “gap (inactive) level”. Accordingly, the following elements can be shallow donors (i.e., donate electrons even at RT): (i) trivalent elements at the substitutional (Mg) site (namely Al, Mn, Fe, and Co) and (ii) cationic elements in the interlayer (namely Na, K, Cu, and Ag). The potential shallow acceptors are (i) monovalent elements at the Mg site (namely Na and K) and (ii) anionic elements in the interlayer (namely F). However, it is not certain that Na and K can occupy the Mg site. Mg vacancies would also be shallow acceptors.

To control the conduction type and conductivity, it is necessary to control the site selectivity of impurity atoms. For Cu-doped Mg(OH)_2_, our research group has found that the conduction type of Cu-doped Mg(OH)_2_ depends on the conditions of electrochemical deposition. This could be due to the fact that the site occupation of Cu is controlled through deposition parameters. In addition, to control the conductivity, the incorporation of compensating ions into the interlayer needs to be suppressed. For example, when Al or Fe is doped as a donor, the incorporation of interlayer anions should be suppressed.

As noted in the introduction, Mg(OH)_2_ has already been used for solar cells. If conductivity can be controlled by impurity doping, it may be possible to fabricate a pn homojunction based on Mg(OH)_2_. Then, Mg(OH)_2_ could be applied in electronic devices such as TFTs. Moreover, with adequate conductivity, Mg(OH)_2_ may be used for transparent electrodes. As previously discussed, the conductivity of heavily C-doped Mg(OH)_2_ is high enough for electrodes. For applications in optoelectronics, the optical absorption and emission properties of Mg(OH)_2_ need to be further investigated. Research can also be extended to other metal hydroxides. So far, many kinds of oxides have been investigated as TCO, but comparatively little attention has been paid to hydroxides. Among them, there could be potential candidates applicable to electronics, such as transparent conductive hydroxides (TCHO). Finally, it should be noted that studies on impurity doping in Mg(OH)_2_ have been limited to Cu and C, either experimentally or theoretically. The validity of the present calculations should be assessed by experiments and calculations based on other methods. Future studies of our group will involve fabrication of impurity-doped Mg(OH)_2_ via wet chemical techniques, which will allow us to verify the present results and demonstrate the possibility of applications for electronic devices.

## 4. Conclusions

The energy levels of impurities introduced into Mg(OH)_2_ were predicted by first-principles calculations. According to the results, trivalent cations at the substitutional site and cation atoms at the interlayer site are possible shallow donors. An interlayer halogen atom can be a shallow acceptor. The introduction of a large amount of C could lead to metallic conduction due to the formation of a 2D structure in the interlayer. Impurity doping could give Mg(OH)_2_ n-type or p-type conductivity, allowing it to be used for electronic applications, especially in transparent electronics.

## Figures and Tables

**Table 1:** Impurity elements considered in the calculation.

Site	Element
Substitutional	Na, K, Ca, Mn, Fe, Co, Ni, Cu, Zn, Al, Si, Sn, C
Interlayer	Na, K, Cu, Ag, F, Cl, C

**Table 2:** Energy positions (eV) of deep levels introduced by impurities.

	VOH	s-Cu	s-Si	s-Sn	s-Ni	i-C	s-C	O–C
Unoccupied	-	-	-	-	-	Ec − 0.8	-	-
Partially occupied	Ec − 1.0	Ev + 1.5	-	-	-	-	-	Ec − 1.0
Occupied	-	-	Ec − 1.1	Ev + 2.3	Ev + 1.6 Ec − 1.1	Ev + 1.8	Ev + 0.8	-

**Table 3:** Predicted character of impurity atoms in Mg(OH)_2_.

Character		Element
Shallow (E_a_ < 0.5 eV)	donor	s-Al, s-Mn, s-Fe, s-Co, i-Na, i-K, i-Cu, i-Ag
	acceptor	V_Mg_, i-F, s-Na, s-K
Deep (0.5 eV < E_a_ < 1.0 eV)	donor	V_OH_
	acceptor	i-Cl
Deep (inactive) level		s-Cu, s-Ni, s-Si, s-Sn, C
No gap level		s-Ca, s-Zn
